# The Role of Community Pharmacies in Creating Harm Reduction Infrastructure to Address the US Opioid Crisis

**DOI:** 10.5888/pcd23.260181

**Published:** 2026-10-08

**Authors:** Isaiah Ratz, Landon Woinarowicz, Heidi Eukel

**Affiliations:** 1 Department of Pharmacy Practice, North Dakota State University, Fargo, North Dakota

The US continues to experience the harmful effects of the opioid epidemic, including those related to overdose and injection-related infectious diseases. More than 80,000 deaths due to opioid overdose were reported in 2024, underscoring the persistent impact of the opioid epidemic nationwide (1). Injection drug use contributes to the transmission of HIV and hepatitis C. For example, a rural Indiana outbreak investigation identified 181 HIV infections in a network of people who injected drugs, 167 (92.3%) of whom were co-infected with the hepatitis C virus (2). Broader analyses further indicated that rural counties with high rates of opioid prescribing and low socioeconomic status often experienced overlapping clusters of overdose deaths and indicators of vulnerability to HIV and hepatitis C (3). These findings illustrate the dual burden of overdose and injection-related infectious diseases in the US, particularly in rural communities.

To address this issue, harm reduction strategies such as naloxone distribution, syringe service programs, and infectious disease testing have increasingly been implemented in recent years. Community pharmacies are highly accessible health care settings that can expand the reach of overdose prevention and infectious disease mitigation strategies. Because pharmacists routinely interact with patients receiving opioid medications and others at risk for injection-related harms, pharmacies are positioned to deliver harm reduction interventions and serve as access points for public health prevention.

Despite the accessibility of community pharmacies, access to harm reduction services in these pharmacy settings is inconsistent, and few community pharmacies focus on harm reduction initiatives. A study of pharmacies in rural Georgia found that only 55% reported having naloxone in stock, suggesting that overdose prevention tools are not uniformly available even in settings positioned to deliver them (4). Similar disparities in geographic access exist for syringe service programs. National analyses indicated that many people with hepatitis C lived substantial distances from these programs, that most resided more than 10 miles from the nearest site, and that rural residents faced the greatest travel burdens (5). These findings illustrate how availability and geographic barriers can limit access to harm reduction services, particularly in rural communities where prevention resources and alternative services are often scarce. This underscores the opportunity to create an infrastructure of harm reduction programming in community pharmacies.

## Barriers to Pharmacy-Based Harm Reduction Programming

Gaps in access to harm reduction services persist because financial, operational, and social challenges hinder consistent implementation of harm reduction strategies. These challenges were seen in studies of pharmacists in New Mexico, which identified financial and operational challenges affecting distribution of naloxone, including high out-of-pocket costs for patients, limited time for counseling by the pharmacist, and lack of reimbursement for naloxone-related services (6). Stigma is another challenge: some pharmacists reported concerns that dispensing naloxone might promote opioid misuse or attract undesirable clientele (6).

Access to sterile syringes is a core component of harm reduction, yet financial, operational, and social challenges continue to impede access. Even when policies permit nonprescription syringe sales, implementation may be inconsistent. After Georgia lifted its ban on nonprescription syringe sales, a survey of pharmacies found that many still refused to sell syringes without a medical indication, often citing security concerns, corporate policies, or the belief that syringe access could encourage drug use (7). Many pharmacists were unaware that state law had changed, highlighting how gaps in communication and in policy-related training can hinder adoption of harm reduction practices. Together, these financial, operational, and social barriers contribute to uneven implementation of harm reduction services and may disproportionately affect rural and underserved populations that rely heavily on community pharmacies for prevention resources (6,8).

Historically, harm reduction efforts to address the US opioid crisis have been delivered through public health departments, community-based programs, and increasingly through community pharmacies. These efforts increase access to naloxone and sterile syringes while also serving as entry points for additional health services such as infectious disease testing, vaccination, and links to treatment. Collectively, these efforts have demonstrated that expanding access to harm reduction resources can reduce mortality rates due to overdose and mitigate injection-related health risks at the population level (3,5,8,9).

Despite demonstrated benefits, current approaches to harm reduction are limited by geographic disparities, implementation challenges, and stigma. The availability of naloxone varies widely across communities, with substantially lower availability reported in many rural areas (4). Stigma, perceived legal risks, and corporate policies also contribute to restrictive pharmacy practices despite laws permitting nonprescription syringe sales. Gaps in training further complicate implementation; many pharmacists report feeling unprepared to identify patients at risk for overdose or to provide naloxone counseling (6). Ultimately, these limitations mean that legal authorization alone does not guarantee consistent implementation of harm reduction strategies across health care and community settings.

## Integrating Harm Reduction Into Routine Community Pharmacy Practice

To address these gaps in services and capitalize on the opportunities available, harm reduction must be incorporated into routine community pharmacy practice. A shift in community pharmacy practice is needed to move from passive dispensing to proactive, integrated harm reduction services (Table). Offering naloxone routinely based on risk indicators rather than patient request allows overdose prevention to occur as a standard component of care. This approach reduces missed opportunities for intervention and creates a consistent expectation that naloxone is part of safe opioid use. When these services were supported by defined workflows and clear roles, pharmacists reported more feasibility and more willingness to provide harm reduction interventions (10).

**Table:** Transition From Passive Dispensing to Harm Reduction Infrastructure in US Community Pharmacies to Address the Opioid Crisis

Domain	Current State	Integrated Model
Naloxone	Patient-request driven	Proactive, protocol-based
Syringe access	Variable, unclear workflow	Standardized service with disposal
Referrals	Ad hoc	Formalized linkage pathways
Pharmacist role	Product-focused	Prevention-focused
Public health integration	Minimal	Bidirectional partnership
Rural reach	Limited	High-access distribution node

Community pharmacy–based syringe access and disposal programs present a similar opportunity for extension of existing service models. These programs can be incorporated into established dispensing processes in the same way immunizations and point-of-care testing have been. Community pharmacies can thereby function as reliable access points for safer injection supplies while also addressing community concerns related to improperly discarded syringes (Table).

Standardized referral pathways further expand the role of the community pharmacy from product distribution to a point of entry into the continuum of care. Structured referral pathways expand the role of the community pharmacy beyond product distribution by connecting patients to medications for opioid use disorder, infectious disease testing, and social services, creating a coordinated pathway for prevention and treatment (Table). Workforce preparation is necessary for these services to occur consistently. Pharmacist training that focuses on harm reduction principles, stigma reduction, and person-centered communication can improve pharmacist confidence and facilitate integration of harm reduction services into routine workflow (10). These models are most sustainable when developed in partnership with public health agencies and supported through reimbursement mechanisms that recognize prevention services as clinical care (10).

People who inject drugs would benefit from the expansion of low-barrier services into community pharmacy settings. These locations offer multiple opportunities for engagement through routine prescription dispensing, medication counseling, immunizations, and other pharmacy services and are geographically distributed. Prevention services can thus occur in familiar and accessible settings without requiring engagement in traditional health care systems. Rural communities are likely to experience the greatest benefit when community pharmacies engage in harm reduction efforts. In many of these areas, the community pharmacy is the most consistently available health care resource. The broad geographic distribution of community pharmacies, combined with extended hours, walk-in access, and established patient relationships, positions them to function as localized prevention infrastructure, particularly where stand-alone harm reduction programs may be limited or unavailable (9). These characteristics allow community pharmacies to serve patients who may otherwise experience barriers to accessing harm reduction services. When stand-alone harm reduction programs are not adequately staffed, they may not be accessible to patients. In addition, patients may face transportation challenges when accessing these programs.

Community and independent pharmacies (ie, privately owned pharmacies that operate independently of national chain organizations) benefit from integration into local public health strategies and the development of sustainable harm reduction services that align with prevention-focused care. Public health systems benefit from increased reach without the need to establish new infrastructure, allowing population-level interventions to be delivered through an existing network that already maintains routine contact with the community. The incorporation of harm reduction into routine community pharmacy practice represents a transition from episodic intervention to standardized prevention. As naloxone distribution, syringe access and disposal services, and structured referral processes become expected components of care, these services will no longer be dependent on individual pharmacist initiative.

## Working Toward Sustainability

At the population level, meaningful reductions in mortality rates due to opioid overdose require a scale of naloxone distribution and saturation that exceeds the capacity of traditional program models. National estimates have demonstrated that extensive and sustained coverage is necessary to meet community need, a threshold that can be reached only through widely distributed access points such as community pharmacies (9). Repeated contact in these settings between pharmacists and community members allows prevention to occur earlier and more consistently.

Long-term sustainability depends on alignment across policy, payment, workforce training, and public health collaboration. When these elements are coordinated, community pharmacies function as permanent components of harm reduction infrastructure rather than time-limited pilot sites in addressing the US opioid crisis. This alignment increases the availability of prevention services, improves equity in access to these services, and strengthens the capacity of health systems to respond to evolving trends in substance use and infectious disease.
